# Longitudinal Network Relationships Between Symptoms of Deviant Peer Affiliation and Internet Gaming Disorder in Adolescents: Prospective Cohort Study

**DOI:** 10.2196/72543

**Published:** 2025-06-13

**Authors:** Tingting Gao, Fengtong Qian, Rui Li, Yimeng Lyu, Yingying Su

**Affiliations:** 1 Department of Social Medicine and Health Management, School of Public Health, Cheeloo College of Medicine, Shandong University, Jinan, China; 2 NHC Key Lab of Health Economics and Policy Research, Shandong University, Jinan, China; 3 Center for Health Management and Policy Research, Shandong University (Shandong Provincial Key New Think Tank), Jinan, China; 4 School of Public Health, Wannan Medical College, Wuhu, China; 5 School of Public Health and Emergency Management, Southern University of Science and Technology, 1088 Xueyuan Avenue, Shenzhen, 518055, China, 86 075588010114

**Keywords:** deviant peer affiliation, internet gaming disorder, cross-lagged panel network, adolescents, longitudinal design

## Abstract

**Background:**

Previous studies have revealed that deviant peer affiliation (DPA) and internet gaming disorder (IGD) are highly related and often co-occur. Nevertheless, the dynamic interactions among these symptoms, their bidirectional effects, and the underlying mechanisms contributing to their persistence remain poorly understood. Most existing research relies on cross-sectional designs or examines aggregated scores, limiting the ability to detect time-dependent symptom interactions. In particular, prior studies overlook the complex bidirectional relationships between specific IGD and DPA symptoms over time. As adolescence is a developmental period marked by rapid changes in peer relationships and behavioral regulation, distinguishing temporal associations between specific DPA and IGD symptoms at both the within- and between-person levels is important for clarifying mechanisms of their co-occurrence and informing personalized intervention strategies.

**Objective:**

This study aims to examine the temporal and directional relationships between DPA and IGD at the symptom level and differentiate within-person dynamics from between-person differences in their co-occurrence. By focusing on specific symptom-level interactions, this study aims to unravel whether changes in one behavior drive changes in the other over time and to clarify whether their relationship is more influenced by individual changes or stable characteristics.

**Methods:**

Data were drawn from a prospective cohort study including a representative sample of 3296 adolescents. Cross-lagged panel networks analysis was used to explore the directional and temporal associations between individual symptoms of DPA and IGD across time, disentangling within-person dynamics from between-person differences as well as identifying the most influential symptoms that drive their interactions over time.

**Results:**

At the within-person level, several IGD symptoms exhibited predictive effects on subsequent DPA symptoms. Specifically, jeopardized school or work performance due to gaming (IGD10) predicted deviant peer behaviors, including friends’ smoking (DPA1; β=.403) and friends’ stealing (DPA4; β=.393). In addition, losing significant relationships due to gaming (IGD9) strongly predicted friends’ misbehavior (DPA6; β=*.*475*).* However, no significant between-person associations were observed, suggesting that the co-occurrence of IGD and DPA is not primarily explained by stable trait-level differences, but rather by dynamic, time-varying processes occurring within individuals.

**Conclusions:**

Our findings provided valuable insights into the complex relationships between DPA and IGD symptoms, highlighting that the IGD-DPA relationship is primarily driven by within-person dynamic changes rather than stable trait-level differences. This suggests that interventions aimed at reducing IGD risk should focus on addressing the dynamic, time-dependent interactions between gaming behaviors and peer relationships. Prevention programs should focus on healthy peer relationships and adaptive coping strategies to reduce IGD risk and its social consequences. Targeted interventions could disrupt the cycle of maladaptive behavior by focusing on improving both peer and social functioning during critical developmental periods, ultimately preventing the exacerbation of these behavioral issues over time.

## Introduction

Internet gaming disorder (IGD) is an important form of internet addiction (IA) [1], characterized by repeated and uncontrollable use of the internet for gaming, often in multiplayer environments [2]. Noteworthy, it is reported that the pooled prevalence of IGD among adolescents was between 4.6% [3] and 8.8% [4], raising concerns about its psychological and social consequences. IGD is associated with diminished academic performance, disrupted sleep patterns, impaired relationships, and increased risk of depression and anxiety [5]. Many adolescent internet gamers with IGD are reluctant to seek professional help, making early treatment hard to attain [6]. Therefore, preventing IGD is of particular importance.

Among various environmental factors contributing to IGD, peer influences play a crucial role in shaping adolescent behaviors [7]. Adolescence is a developmental stage characterized by increased peer influence, particularly from those engaged in maladaptive behaviors, a phenomenon known as deviant peer affiliation (DPA) [8]. Prior studies have suggested that adolescents who interact with deviant peers are more susceptible to IGD due to observational learning and reinforcement of gaming behaviors within these groups [9].

The relationship between DPA and IGD can be explained through multiple theoretical perspectives. Differential association theory posits that frequent interaction with peers who excessively engage in internet gaming may amplify adolescents’ exposure to deviant beliefs and behavioral patterns, subsequently encouraging their involvement in problematic internet gaming activities [10]. Group socialization theory further suggests that peer groups shape individual behaviors through social comparison and conformity mechanisms [11]. When diverse groups interact, group contrast effects emphasize differences and downplay similarities, which fosters a sense of group identity but can also lead to an “us versus them” attitude [12]. Within groups, assimilation processes promote conformity, while differentiation mechanisms drive social comparisons, influencing individuals’ identities as they align with group norms to prevent exclusion [13]. In addition, the social network theory highlights how peer selection processes contribute to IGD development. Adolescents with IGD may experience weakened connections to mainstream peer groups, increasing the likelihood of affiliating with deviant peers. Through homophily selection, individuals tend to associate with peers who share similar behavioral patterns, reinforcing IGD-related behaviors [14-17]. Meanwhile, default selection occurs when adolescents join deviant peer groups due to limited social alternatives, further intensifying problematic gaming behaviors [18,19].

While these theoretical perspectives support the relationship between DPA and IGD, empirical findings remain inconsistent. Some studies suggest that not all deviant peer relationships contribute to IGD. For certain adolescents, gaming may serve as a form of social bonding rather than deviant behavior [20]. In addition, cultural factors and contextual influences may impact this relationship, leading to variability in its strength and directionality across different social environments. These inconsistencies highlight the need for advanced methodological approaches that can capture the dynamic and bidirectional nature of the DPA-IGD relationship. Traditional methods, which often rely on static or between-person analyses, may overlook the symptom-level interactions and temporal dynamics that characterize these behaviors.

To address these complexities, network analysis provides a valuable methodological approach by offering a visual representation of dynamic relationships by mapping interconnected nodes and edges to illustrate symptom-level interactions between DPA and IGD [21]. In particular, cross-lagged panel networks (CLPNs) help clarify the directionality of relationships, allowing for a more rigorous and logical analysis of examining how symptoms evolve over time [22]. Furthermore, researchers emphasize the necessity of distinguishing between within-individual and between-individual effects [23,24]. Specifically, within-person effects capture temporal dynamics and interrelationships of a phenomenon within an individual, whereas between-person effects investigate the consistent differences in how a phenomenon is experienced across different individuals in a population [25]. CLPN provides a unique advantage by simultaneously modeling both within-person and between-person effects. It thus allows for disentangling the dynamic, symptom-level interactions within individuals over time (within-person effects) from the stable, trait-like differences across individuals (between-person effects). This dual focus is critical for understanding the relationship between DPA and IGD, as it captures both the temporal evolution of symptoms and the broader population-level patterns. By applying the CLPN approach, both the temporal and individual dimensions of the DPA-IGD relationship can be examined simultaneously, overcoming the limitations of traditional analytical methods.

In summary, although prior research has documented reciprocal associations between DPA and IGD, studies examining the underlying mechanisms driving symptom dynamics and the temporal evolution of these relationships remain limited. Notably, few studies have systematically distinguished between within-person and between-person effects in this context. Therefore, this study aims to identify key symptoms that influence the co-occurrence of IGD and DPA in adolescents and to differentiate between within-person and between-person effects. The study findings are expected to provide novel insights into the mechanisms underlying IGD and DPA progression, contributing to the development of targeted prevention and intervention strategies for at-risk adolescents.

## Methods

### Study Design and Participants

Using a longitudinal cohort design, we recruited students from two middle schools and a senior high school in Shandong, China. Data were collected at three time points: Time 1 (T1; November 2021, mean age: 15.17, SD 1.44 y), Time 2 (T2; May 2022, mean age: 16.19, SD 1.43 y), and Time 3 (T3; May 2023, mean age: 17.50, SD 1.18 y). In this study, we focused on T1 and T3 data to examine the long-term developmental dynamics of DPA and IGD over an 18-month period. Time 1 and Time 3 were more suitable for capturing the broader and stable trajectory of these behaviors. In addition, by focusing on these 2 key time points, we aimed to reduce model complexity and mitigate the risk of overfitting, ensuring the analysis focused on the most relevant time-dependent interactions.

### Measures

#### Internet Gaming Disorder

The symptoms of IGD were assessed using a self-reported 10-Item Internet Gaming Disorder Test (IGDT-10) [26,27]. The IGDT-10 is based on the *Diagnostic and Statistical Manual of Mental Disorders, Fifth Edition* (*DSM-5*) diagnostic criteria for IGD. Response options ranged from 0 (never) to 2 (often), with higher scores representing greater severity of problematic internet game use. Higher total scores represent more severe IGD symptoms. The internal consistency of the scale, as measured by Cronbach α coefficient, was 0.83 at T1 and 0.89 at T3, indicating high reliability and consistency across time.

#### Deviant Peer Affiliation

The 8-item DPA scale was used to examine adolescents’ affiliation with deviant peers [8,28]. The items assessed how many of the respondents’ peers had engaged in deviant behaviors over the past 12 months. Responses were rated on a 5-point scale, ranging from 1 (none) to 5 (all). Higher scores reflected higher levels of DPA. The measure demonstrated good reliability in this study (T1: α=.87; T3: α=.88).

### Statistical Analyses

This study employed cross-sectional and longitudinal network analyses to investigate the dynamic relationships between DPA and IGD symptoms. In particular, we examined both their contemporaneous and temporal interactions, differentiating between within- and between-person effects.

### Cross-Sectional Network Analysis

To explore the contemporaneous relationships between DPA and IGD symptoms, we constructed cross-sectional networks using a Gaussian graphical model combined with the graphical Least Absolute Shrinkage and Selection Operator method and the extended Bayesian information criterion for model selection. This technique regularized regression weights, which reduced smaller coefficients to zero, leading to a more interpretable model. The partial correlation matrices were computed to capture direct connections between symptoms, controlling for spurious relationships. We also evaluated centrality indices to identify the most influential symptoms within each network.

### Longitudinal Network Analysis: Within- Versus Between-Person Effects

To examine the dynamic interplay between DPA and IGD symptoms at the individual level, we employed a CLPN that integrates network modeling with cross-lagged panel modeling. This approach enables individual items to influence one another over time, utilizing two-wave panel data to assess both cross-lagged effects (ie, the impact of a symptom at time point 1 on another symptom at time point 2) and autoregressive effects (ie, the impact of a symptom at time point 1 on itself at time point 2). To estimate the CLPN, we computed autoregressive and cross-lagged coefficients with a series of regularized regressions, controlling for age and residence. Regularized regression was performed using the R (R Core Team) package “glmnet” [29]. In addition, we computed two key measures to evaluate network centrality within these networks: in-expected influence, which reflects the degree to which a symptom is influenced by others, and out-expected influence, which indicates the impact of a symptom on predicting others in future time [30].

In addition, to differentiate between temporal inferences at the between-person and within-person levels, we conducted longitudinal network analysis to investigate the interrelations of nodes using a graphical vector autoregression model for panel data with the “psychometrics” R package [31]. The within-person analysis concentrated on the fluctuation of symptoms over time within the same individual, examining how one symptom might influence another across time. This approach enabled us to identify the most influential symptoms in the DPA-IGD network, providing insights into how changes in deviant peer behaviors impact IGD symptoms and vice versa. On the other hand, the between-person analysis focused on individual differences in symptom severity, capturing the variability in symptom relationships across the sample. This allowed us to investigate whether variations in DPA were associated with differences in IGD symptoms among individuals. Together, these analytical levels provide a robust framework for understanding both the temporal dynamics and individual differences in the DPA-IGD relationship. In addition, a regularization-based model selection approach was used to simplify the network structure for better interpretability. This method produced an interindividual network that represented the partial correlations of node means over time [32]. Nonparametric bootstrapping was used to evaluate edge stability, while case-drop bootstrapping was used to assess the centrality stability. Specifically, edge stability was determined by examining the frequency of edges that maintained or exceeded a correlation stability (CS) coefficient greater than 0.5 across bootstrapping iterations. A CS value above 0.5 is commonly considered to indicate reliable edge consistency across samples. For centrality stability, a case-dropping bootstrap procedure was used to calculate the CS, which estimates the maximum proportion of cases that can be removed while retaining a correlation of at least 0.7 with the original centrality estimates. In accordance with established recommendations, a correlation stability coefficient above 0.5 was interpreted as evidence of sufficient centrality stability.

### Missing Data

Little’s test was used to determine whether the missing data followed a missing completely at random pattern. To retain as much information as possible, the expectation-maximization method was used to impute missing data. The expectation-maximization method is considered effective for generating unbiased parameter estimates under both missing at random and missing completely at random conditions [31,33]. All data analyses were conducted using R version 4.1.1.

### Ethical Considerations

After obtaining approval (LL20210102) from the Research Ethics Committee at the School of Public Health, Shandong University, we began the recruitment process for this study. All participants, including minors, as well as their parents or legal guardians, provided written informed consent prior to participation. The consent process ensured that participation was voluntary, with the right to withdraw from the study at any time without penalty.

All participant data were fully anonymized to protect privacy and confidentiality. Paper-based questionnaires were completed in a controlled setting under the supervision of trained research assistants. Anonymized data were securely stored, with access restricted to authorized research personnel only.

No monetary or material compensation was provided to participants. Participation was entirely voluntary and conducted during regular school hours, minimizing the burden and ensuring fairness.

## Results

**Figure 1.**
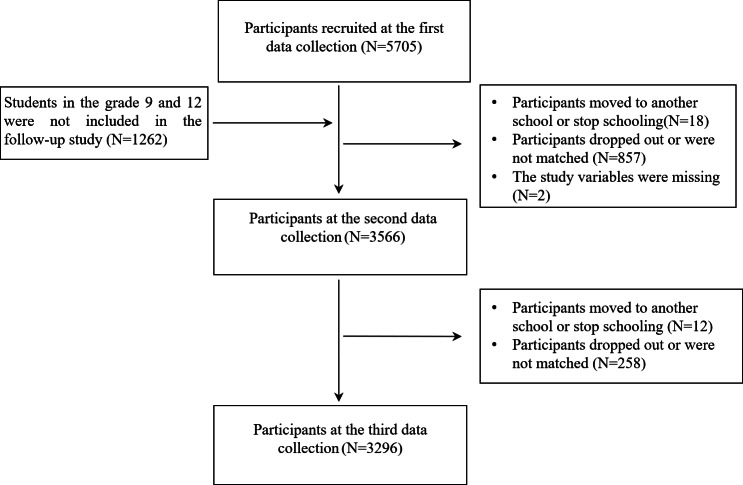
Flowchart of participants included in the analysis.

This study cohort comprised 3296 participants, of whom 54% (=1795) were females and 66% (n=2171) were from rural areas. The grade distribution at baseline was as follows: 38% (n=1243) in Grade 11, 35% (n=1166) in Grade 10, 14% (n=445) in Grade 8, and 13% (n=442) in Grade 7. The detailed sampling procedure is illustrated in Figure 1.

**Figure 2.**
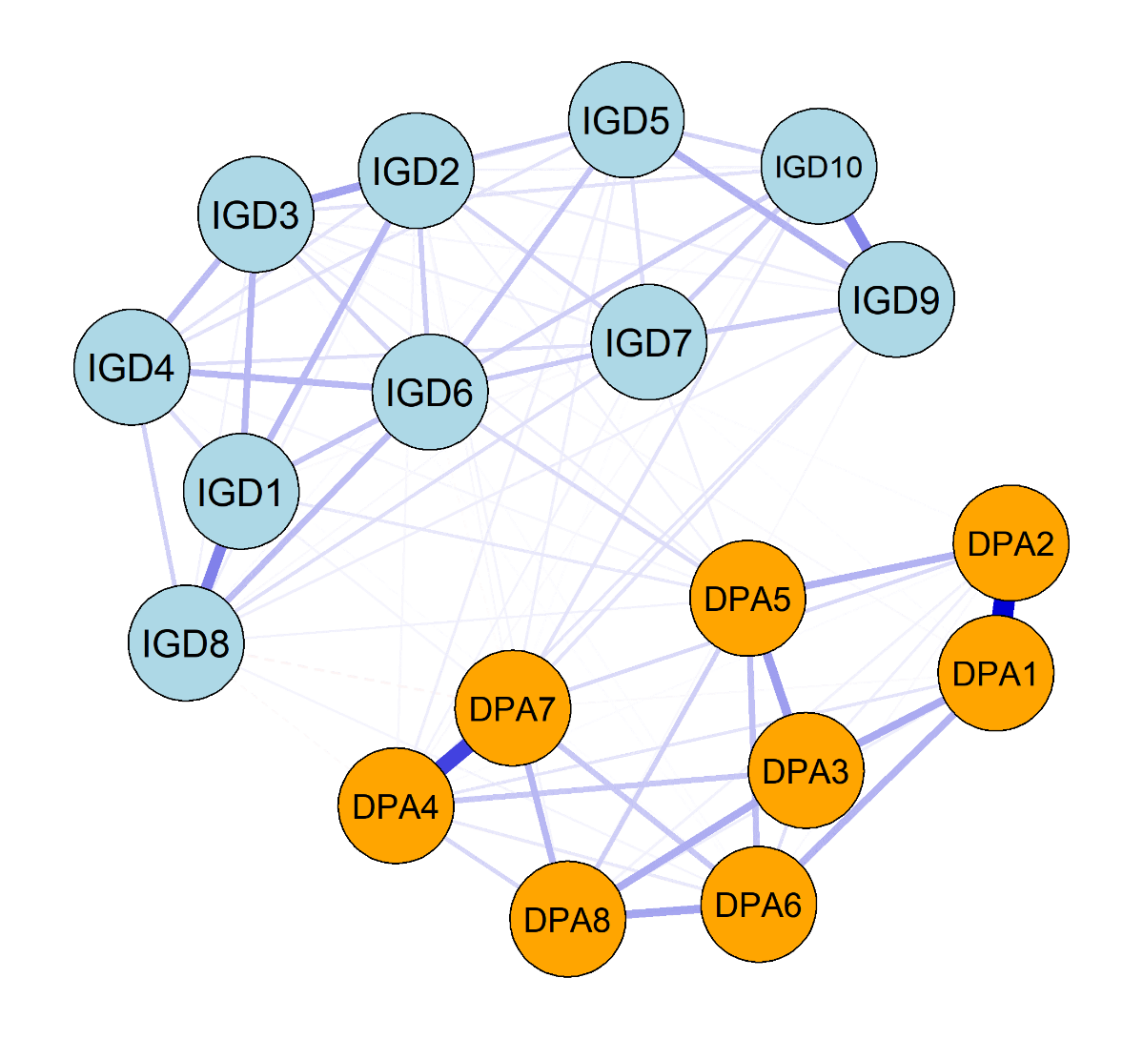
The cross-sectional network of deviant peer affiliation and internet gaming disorder for adolescents. DPA: deviant peer affiliation; IGD: internet gaming disorder.

The strongest positive association was found between friends’ alcohol use (DPA2) and friends’ smoking (DPA1; β=.599) in the cross-sectional network analysis. Furthermore, significant associations were observed between friends’ stealing (DPA4) and friends’ skipping school (DPA7; β=.444), as well as between fantasizing about gaming (IGD1) and playing games to relieve negative emotions (IGD8; β=.296). Refer to Figure 2 for a visual representation of these findings.

Figure S1 in Multimedia Appendix 1 showed that gaming, despite negative consequences (IGD6), had the highest strength centrality. The strength CS coefficient (CS=0.750, 95%CI: 0.672-1.000) indicates that the network has high stability and reliability.

**Figure 3.**
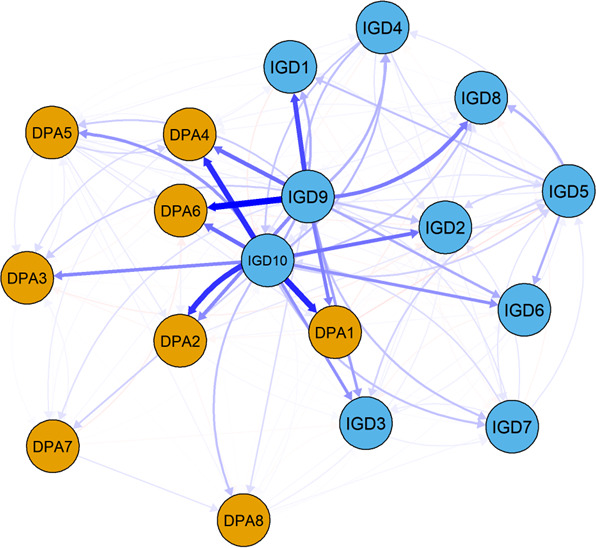
The cross-lagged panel network of deviant peer affiliation and internet gaming disorder for adolescents from Time 1 to Time 3. DPA: deviant peer affiliation; IGD: internet gaming disorder.

Figure 3 illustrates the T1→T3 CLPN, where directed edges depicted the longitudinal relationships between the symptoms of DPA and IGD. The three strongest cross-lagged associations observed were: losing relationships due to gaming (IGD9) predicting friends’ misbehaving (DPA6; β=.475), jeopardized school or work performance due to gaming (IGD10), predicting friends’ smoking (DPA1; β=.403), as well as friends’ stealing (DPA4; β=.393). These findings suggest that negative consequences associated with excessive gaming, particularly impaired social and academic or work functioning, contribute significantly to deviant peer behaviors over time.

The centrality estimates for the T1→T3 CLPN are shown in Figure S2 in Multimedia Appendix 1. In the T1→T3 network, the symptom of jeopardized school or work performance due to gaming (IGD10) had the highest out-expected influence value, indicating it was the most influential symptom among the others. Friends’ stealing (DPA4) and friends’ misbehaving (DPA6) had the highest in-expected influence values, suggesting that these symptoms were strongly influenced by other symptoms within the model. Additional analyses were performed to assess the stability and reliability of these findings. Figures S3-S5 in Multimedia Appendix 1 display the results of edge weight difference tests and centrality difference tests. The results showed a statistically significant difference between most of the edge weights and node strengths. The narrow-bootstrapped CIs for the edge weights in Figure S6 in Multimedia Appendix 1 indicate that the results are stable and reliable.

Finally, the results of the between-person network for adolescents did not reveal any statistically significant associations between DPA and IGD symptoms. This suggests that, at the between-person level, individual differences in IGD symptoms do not necessarily correspond to variations in deviant peer behaviors.

## Discussion

### Principal Findings

This study employed network analysis to investigate the dynamic relationship between IGD and DPA symptoms across different timescales among adolescents. We identified significant within-person temporal interactions between IGD and DPA symptoms. Specifically, losing significant relationships due to gaming (IGD9) had the strongest cross-lagged predictive effect on friends’ misbehavior (DPA6). In addition, jeopardized school or work performance due to gaming (IGD10) predicted both friends’ smoking (DPA1) and friends’ stealing (DPA4), suggesting that academic failure may increase adolescents’ likelihood of affiliating with deviant peers. However, no significant association was found between IGD and DPA symptoms in the between-person network, indicating that their relationship may primarily manifest as within-person dynamic changes rather than a stable between-person association.

Specifically, in the within-person temporal network, we identified the strongest cross-lagged edge, where losing a significant relationship due to gaming (IGD9) predicted friends’ misbehaving (DPA6). One possible explanation for this finding is that adolescents who engage in excessive gaming may experience reduced socialization in real-life settings, leading to smaller social circles and weakened social ties with nondeviant peers [34]. As a result, these adolescents may seek out new social connections among peers who have faced similar social rejection, including those who exhibit deviant behaviors such as misbehaving with friends or facing disciplinary actions at school [35]. In addition, jeopardized school or work performance due to gaming (IGD10) also strongly predicted friends’ smoking (DPA1) and friends’ stealing (DPA4). This aligns with prior research indicating that academic failure increases the likelihood of affiliation with deviant peers [36]. Co-occurring academic and behavioral issues have also been identified as predictors of DPA, particularly among boys [37]. One possible explanation for this finding is that academic difficulties and problematic gaming behaviors may predispose adolescents to affiliate with deviant peers, including those involved in misconduct, such as misbehavior or theft. Another plausible explanation is that IA itself is a characteristic commonly observed among deviant peers, further reinforcing the association between excessive gaming and other deviant behaviors [38]. Furthermore, our findings highlight jeopardized school or work performance due to gaming (IGD10) as the most influential symptom in the cross-lagged network, suggesting that it plays a key role in shaping the developmental trajectory of both IGD and DPA. This finding is consistent with a previous cross-lagged panel network analysis of problematic mobile phone use and negative emotions, which found that reduced productivity at school, work, or home due to excessive mobile phone use had the highest out-expected influence, indicating its strongest impact over time [39]. In contrast, friends’ stealing (DPA4) and friends’ misbehaving (DPA6) were the most vulnerable to external influences within the network, reinforcing the notion that certain deviant peer behaviors may be particularly sensitive to risk factors associated with IGD.

Beyond these temporal associations, the contemporaneous network also captured strong co-occurring relationships between DPA and IGD. The strongest positive associations were observed between: (1) friends’ alcohol use (DPA2) and smoking (DPA1), (2) friends’ stealing (DPA4) and skipping school (DPA7). Empirical evidence has shown a co-occurrence between alcohol use disorders and smoking [40,41]. In addition, stealing and truancy were identified as core behavioral patterns associated with attention-deficit hyperactivity disorder in a county-wide sample [42]. We also identified a significant association between fantasizing about gaming (IGD1) and playing games to relieve negative emotions (IGD8), which aligns with the compensatory internet use theory [43]. This theory posits that individuals may engage in excessive gaming as a maladaptive coping mechanism to manage stress and alleviate negative emotions. Similar findings have been observed in studies on IA among Chinese college students, where fantasizing about online activities was identified as a key symptom triggering other addictive behaviors [44]. The results also recognized gaming despite negative consequences (IGD6) as the central symptom in the network. This finding reinforces the widely recognized clinical characteristic of over-engagement in gaming despite negative consequences is a key clinical characteristic of IGD [45]. Adolescents with IGD may exhibit decision-making patterns similar to their deviant peers, particularly in situations involving risk-taking behaviors [46,47].

Contrary to our findings in the within-person analysis, no statistically significant relationships were observed between DPA and IGD symptoms in the between-person network. This implies that variations in IGD symptoms among individuals do not inherently correspond to differences in deviant peer behaviors at the between-person level. This suggests that the association between DPA and IGD symptoms is contextually dynamic. Their relationship exhibits stronger dynamic, situational, and bidirectional influences at the within-person level rather than stable trait-level differences across individuals. These findings highlight the importance of tailoring interventions to address situational triggers (eg, immediate peer interactions) rather than relying solely on group-level risk profiles.

Our findings have both theoretical and practical implications. While prior research has mainly focused on cross-sectional analysis across individuals, network methodologies enable the simultaneous exploration of multiple variables within a single individual. In this study, we provide new theoretical insights into the relationship between DPA and IGD by examining differences across between-person contemporaneous, within-person contemporaneous, and within-person temporal networks. From a practical standpoint, our research revealed the temporal and causal links within these complex systems, highlighting the potential need to intervene on multiple symptoms simultaneously. Particularly in adolescents, it is important to prioritize addressing jeopardized school or work performance due to gaming (IGD10) and gaming despite negative consequences (IGD6) in the prevention program. In addition, our findings emphasize the importance of incorporating the strongest edge into intervention strategies, with a particular focus on the predictive effects of one symptom on another, to optimize therapeutic outcomes.

The present study sheds light on the complex relationship between DPA and IGD from a network perspective, contributing valuable insights to the existing literature by revealing their symptom-level pathways, central nodes, and potential targets for intervention in the relationship between DPA and IGD. This study also suggests that their relationship may primarily manifest as within-person dynamic changes rather than stable between-person differences. However, several limitations should be acknowledged. First, the study relies exclusively on adolescents’ self-reported data, which might introduce measurement bias. Future research should incorporate multiple sources of information, such as parents and teachers, to gain a more holistic and contextually grounded understanding of both DPA and IGD. Second, the study sample was limited to Chinese adolescents. To enhance the robustness and generalizability of our findings, it is essential to validate these results across different cultural and demographic populations. Third, while this study offers valuable insights into the potentially causal associations between DPA and IGD symptoms over an 18-month period, it is important to note that altering the time lag could potentially yield different results. Therefore, future research should aim to replicate these findings with different time intervals to explore the potential variations in these results.

### Conclusion

In conclusion, this study offers novel insights into the dynamic and directional relationships between IGD and DPA symptoms among adolescents. Through longitudinal network analysis over an 18-month period, we demonstrated that their co-occurrence is predominantly driven by within-person fluctuations rather than stable between-person traits. Notably, symptoms such as jeopardized school or work performance due to gaming (IGD10) and gaming despite negative consequences (IGD6) emerged as central nodes in the temporal and contemporaneous networks, highlighting their pivotal roles in the developmental progression of IGD and DPA. These findings underscore the need for prevention and intervention strategies that target context-sensitive, symptom-level mechanisms during critical stages of adolescent development, as such approaches may be especially effective in disrupting the reinforcing cycle between gaming problems and deviant peer behaviors.

Future research should further explore the dynamic interplay between IGD and DPA using high-resolution temporal data to capture micro-level fluctuations in real-world contexts. In addition, integrating objective behavioral data, such as digital activity logs or peer nomination methods, can enhance the validity of symptom measurement and provide a more comprehensive view of adolescents’ social environments. In addition, future longitudinal network studies spanning different developmental periods may help reveal how age or contextual factors influence the temporal dynamics and structural patterns of the IGD-DPA relationship.

## Supplementary material

10.2196/72543 Multimedia Appendix 1 Supplementary materials.

## Abbreviations

CLPN: cross-lagged panel network
CS: correlation stability
DPA: deviant peer affiliation
*DSM-5*: *Diagnostic and Statistical Manual of Mental Disorders, Fifth Edition*
IA: internet addiction
IGD: internet gaming disorder
IGDT-10: 10-Item Internet Gaming Disorder Test
