# Supplementary material for: Longitudinal Network Relationships Between Symptoms of Deviant Peer Affiliation and Internet Gaming Disorder in Adolescents: Prospective Cohort Study
Source: J Med Internet Res. 2025 Jun 13;27:e72543. doi: 10.2196/72543 (PMC12180675; doi:10.2196/72543)
Supplement: Multimedia Appendix 1 [file jmir-v27-e72543-s001.docx]

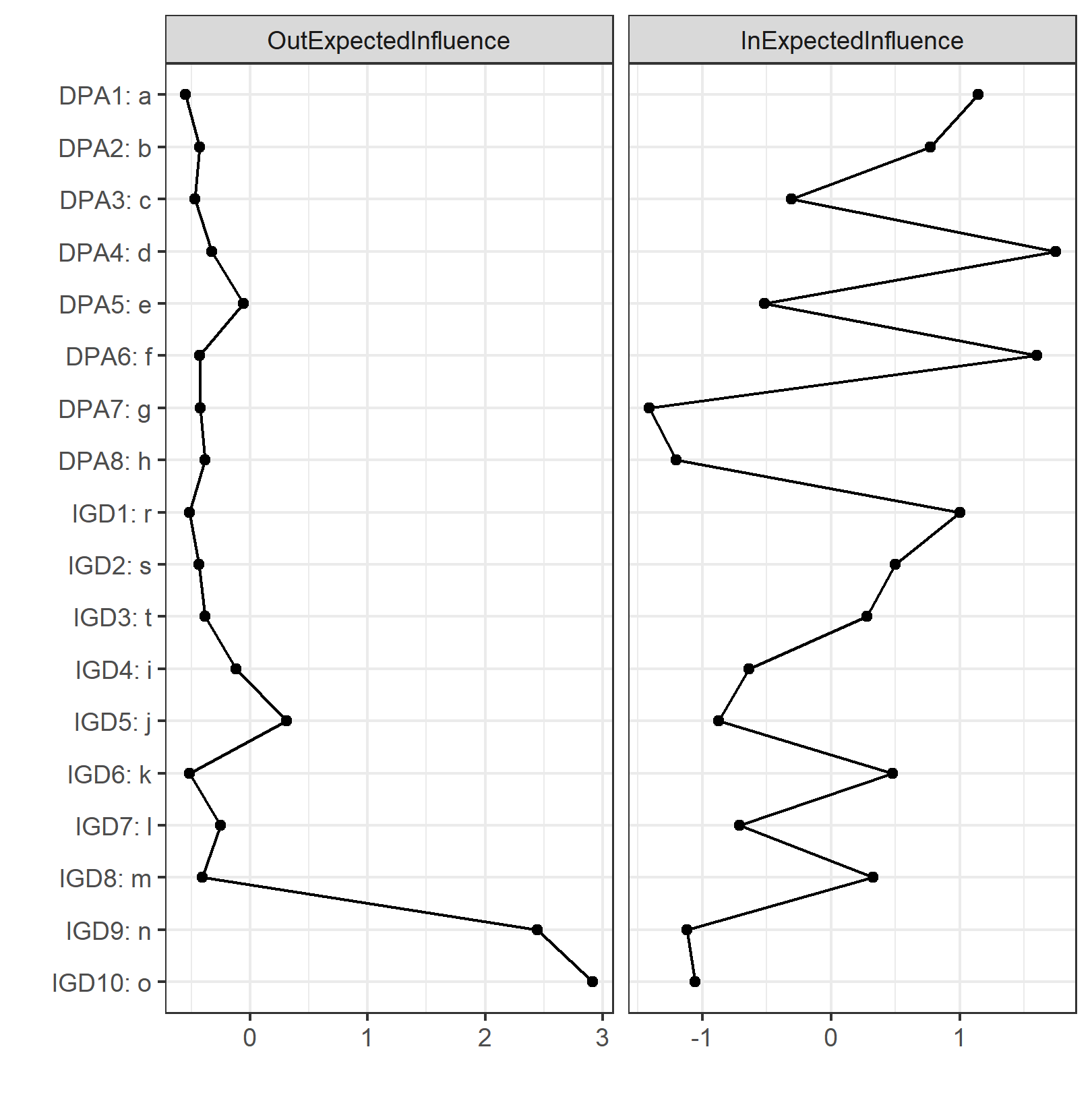


Figure S1. Centrality estimation for T1→T3 network.


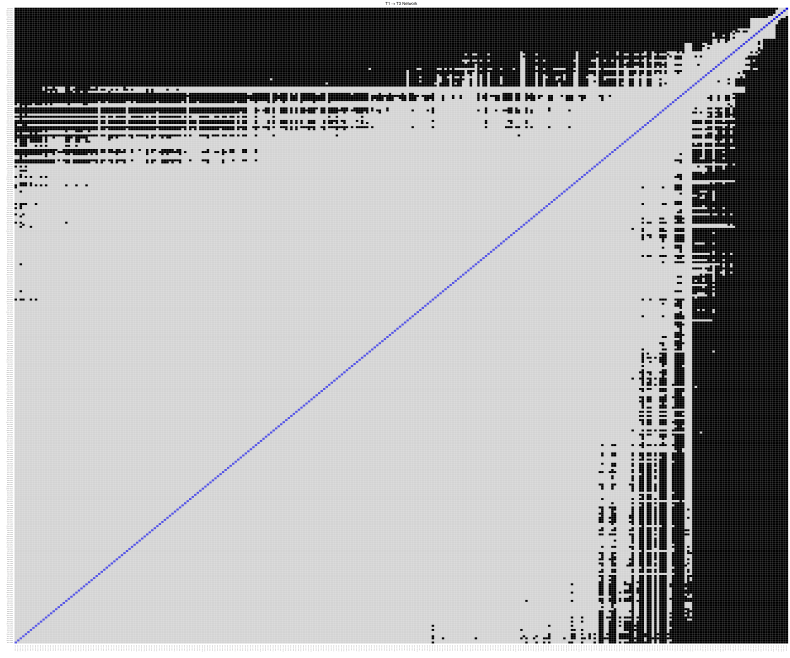


Figure S2. Edge weight difference tests for the networks using T1 to predict T3. Black boxes indicate edges that significantly differ (p < 0.05), and gray boxes indicate edges that do not significantly differ.


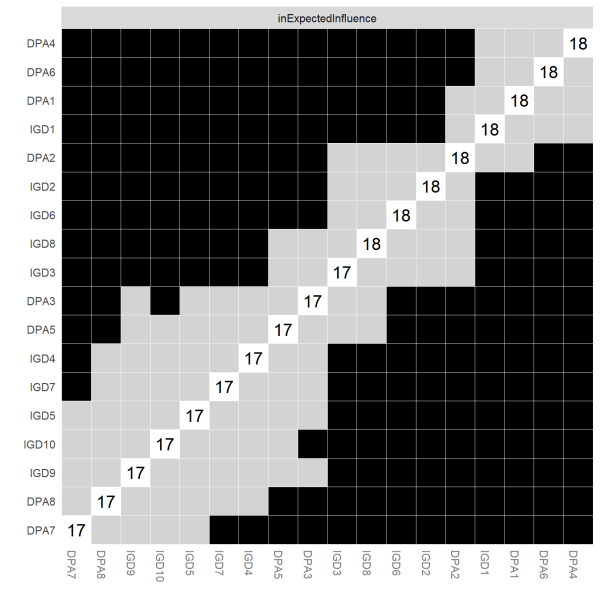

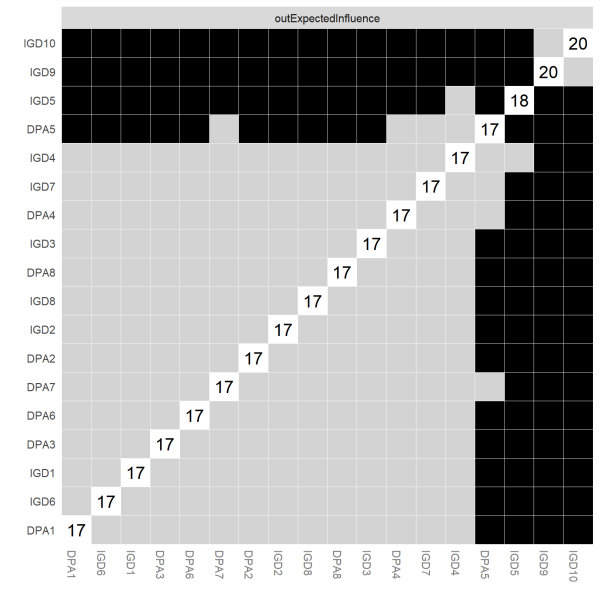


Figure S3. Estimation of edge weight difference by bootstrapped difference test (T1→T3). Bootstrapped difference tests between edge weights in the network. Gray boxes represent the edges do not significantly differ from one-another. Black boxes represent significant difference. Blue boxes in the edge-weight plot correspond to positive correlations.


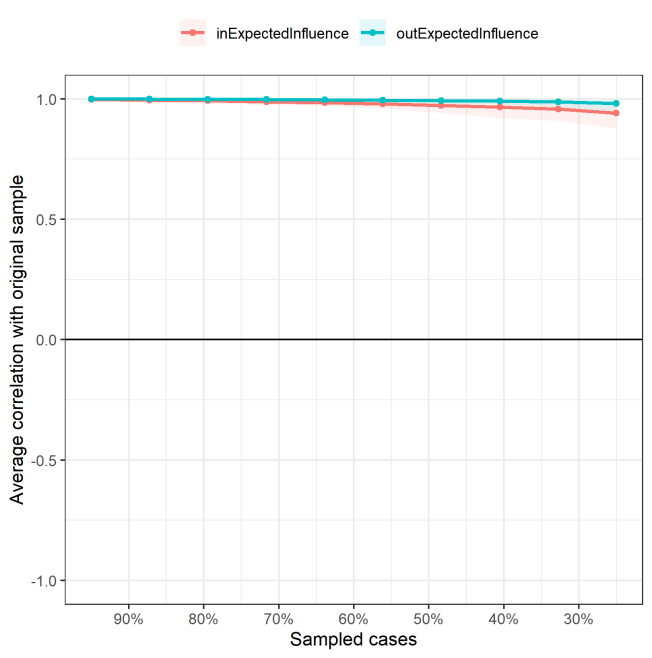


Figure S4. Stability of the centrality indices in the CLPN for T1→T3.


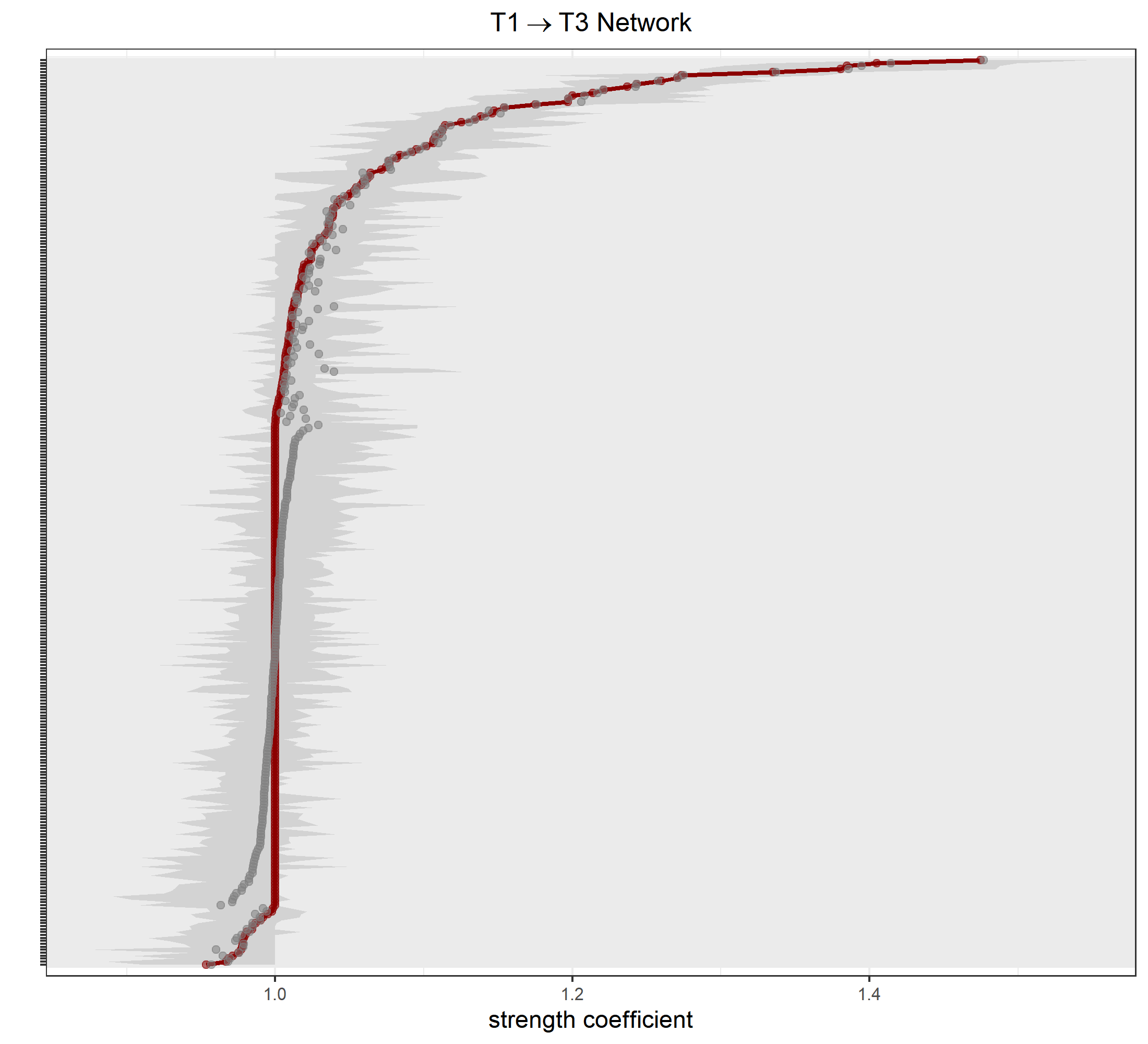


Figure S5. Bootstrapped confidence intervals of edge weights. The red dots indicate the values of each edge weight, ordered from the highest to the lowest values. The gray area represents the 95% Confidence Intervals of edge weights, estimated with the non-parametric bootstrap procedure. Wide intervals indicate lower stability and narrow intervals indicate higher stability.


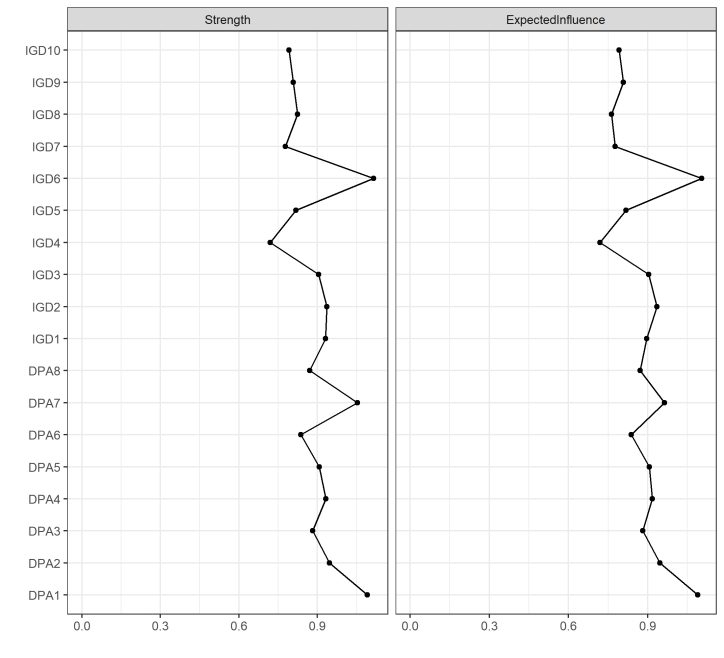


Figure S6. Centrality measures of all symptoms within the network at different stages of the adolescents. The figure shows centrality measures (i.e., strength and expected influence) of all symptoms within the network(z-scores).
